# Randomized Study of Tenapanor Added to Phosphate Binders for Patients With Refractory Hyperphosphatemia

**DOI:** 10.1016/j.ekir.2023.08.003

**Published:** 2023-08-13

**Authors:** Kosaku Nitta, Saki Itoyama, Kazuaki Ikejiri, Jun Kinoshita, Kaoru Nakanishi, Masafumi Fukagawa, Tadao Akizawa

**Affiliations:** 1 Department of Nephrology, Tokyo Women’s Medical University, Tokyo, Japan; 2 Research and Development Division, Kyowa Kirin Co., Ltd., Tokyo, Japan; 3 Division of Nephrology, Endocrinology, and Metabolism, Department of Internal Medicine, Tokai University School of Medicine, Kanagawa, Japan; 4 Division of Nephrology, Department of Medicine, Showa University School of Medicine, Tokyo, Japan

**Keywords:** chronic kidney disease, hemodialysis, hyperphosphatemia, NHE3 transporter, phosphorus absorption inhibition, tenapanor

## Abstract

**Introduction:**

Serum phosphorus management is important for patients with chronic kidney disease on dialysis to reduce the risk of hyperparathyroidism and ectopic vascular calcification. Phosphate binders (PBs) control serum phosphorus levels; however, some patients do not achieve adequate control with existing PBs. The similar mechanisms of action of each PB may limit their ability to lower serum phosphorus levels. Therefore, drugs with novel mechanisms of action that can be added to existing PBs to further lower serum phosphorus levels are desired. Tenapanor, a novel selective inhibitor of intestinal sodium/hydrogen exchanger 3 transporters, decreases passive phosphate absorption in the intestine, thereby decreasing serum phosphorus levels.

**Methods:**

This study evaluated the efficacy and safety of tenapanor treatment with up-titration when added to PBs among Japanese hemodialysis patients with hyperphosphatemia poorly controlled by PBs alone. In total, 169 patients taking PBs whose serum phosphorus level was ≥6.1 and <10.0 mg/dl initiated the 8-week treatment (placebo + PB, *n* = 85; tenapanor + PB, *n* = 84).

**Results:**

The least squares mean change from baseline to week 8 in serum phosphorus level was −0.24 and −2.00 mg/dl in the placebo and tenapanor groups, respectively, with a statistically significant difference between groups (−1.76 mg/dl; *P* < 0.0001). Diarrhea as a treatment-emergent adverse event (TEAE) occurred in 14.1% and 63.1% of patients in the placebo and tenapanor groups, respectively. All diarrhea events were mild or moderate.

**Conclusion:**

Tenapanor added to PBs improved serum phosphorus levels that could not previously be controlled by PBs alone, and no new safety concerns were raised.

## Graphical abstract

**Figure:**
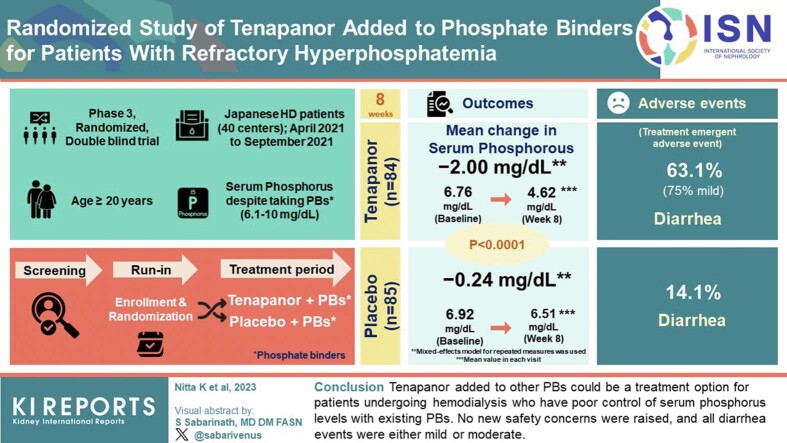

See Commentary on Page 2194

Over 50% of patients with end-stage kidney disease undergoing hemodialysis develop hyperphosphatemia,^1^^,^^2^ one of the most important complications of chronic kidney disease-bone mineral disorder^3^ and a risk factor for cardiovascular events.^4^, ^5^, ^6^ Therefore, appropriate management of serum phosphorus levels^7^^,^^8^ is important for patients undergoing dialysis to prevent these events. In fact, strict control of serum phosphorus levels has been shown to reduce coronary artery calcification in patients on dialysis.^9^

The initial strategies for managing hyperphosphatemia are dietary restriction and adequate dialysis.^3^^,^^10^ When these strategies fail to control serum phosphorus levels sufficiently, PBs are introduced. PBs reduce phosphorus absorption in the intestinal tract by binding directly to phosphate in the intestinal lumen, resulting in fecal excretion. However, existing PBs are limited by adverse effects directly linked to the characteristics of each drug,^10^ and their high pill burden leads to low patient adherence.^11^, ^12^, ^13^

In Japan, as of July 2021, 21.1% of patients on dialysis exceeded the target range of serum phosphorus levels (3.5–6.0 mg/dl) set by the Japanese Society for Dialysis Therapy.^7^ This suggests that, despite multifaceted strategies and the approval of various PBs, serum phosphorus control remains unsuccessful for some patients. Such patients require further treatment, and some use several PBs in combination. However, some patients cannot add any further PBs owing to the heavy pill burden or the occurrence of side effects. In addition, because of the similar mechanisms of action of each PB, some combinations may not provide additional benefits. Therefore, patients with poor serum phosphorus control while receiving PBs have unmet treatment needs. Therefore, they require a drug with a novel mechanism of action that can add to the effect of existing PBs and have fewer side effects and a lower pill burden.

Tenapanor is a selective inhibitor of intestinal sodium/hydrogen exchanger 3 transporters expressed on the intestinal epithelial cells on the luminal side. This action results in decreased passive phosphate absorption through the intercellular space of epithelial cells in the intestines, thereby decreasing the serum phosphorus level.^14^^,^^15^ This is a novel mechanistic approach to treat hyperphosphatemia. Given this novel mechanism of action, adding tenapanor to the existing PB treatment may contribute to serum phosphorus control.

In a US phase 3 trial of patients with poorly controlled serum phosphorus undergoing hemodialysis, tenapanor + PB resulted in a significantly greater mean decrease in serum phosphorus at 4 weeks versus placebo + PB. The most frequently occurring adverse event in the tenapanor group was diarrhea.^16^ In Japan, a phase 2, double-blind, randomized, placebo-controlled 6-week study of hemodialysis patients with refractory hyperphosphatemia also showed that add-on tenapanor significantly reduced serum phosphorus levels versus placebo. Diarrhea as an adverse event occurred more frequently in the tenapanor group than in the placebo group.^17^

The present phase 3 study evaluated the efficacy and safety of tenapanor versus placebo added to existing PBs among Japanese hemodialysis patients with poorly controlled serum phosphorus.

## Methods

### Study Design

**Figure 1:**
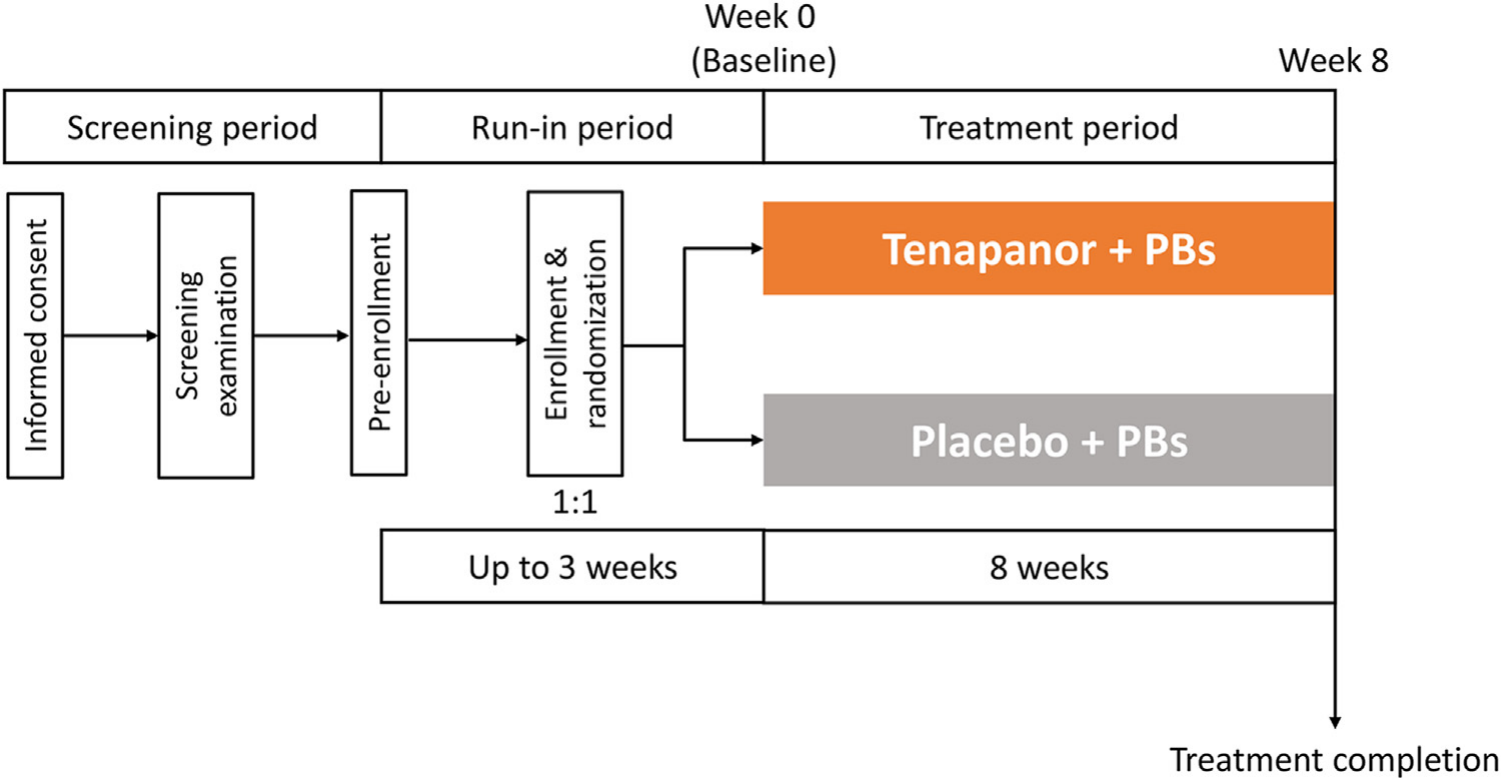
Study design. ∗Subjects were enrolled if they meet the criteria for enrollment, including the criterion for serum phosphorus levels 1 or 2 weeks after the start of the run-in period. PB, phosphate binder.

This was a phase 3, randomized, double-blind, placebo-controlled, parallel-group study, comprising a screening period (including the pre-enrollment), a run-in period (including enrollment and randomization), and a double-blind treatment period of 8 weeks (Figure 1). The study was conducted at 40 facilities in Japan between April 2021 and September 2021.

The Institutional Review Board approved the study protocol, the study adhered to the ethical standards of the Declaration of Helsinki, and all patients provided informed consent before participation. The study was registered at ClinicalTrials.gov under the identifier NCT04766398.

### Patients

The target population consisted of patients aged ≥20 years undergoing hemodialysis, with serum phosphorus levels of ≥6.1 mg/dl and <10.0 mg/dl at screening and 1 or 2 weeks after the run-in period, despite treatment with 1 or more PBs administered before screening. Patients who experienced diarrhea or loose stools (Bristol Stool Form Scale score ≥6) or had ≥3 bowel movements for ≥2 days within 1 week before enrollment were excluded from the study. The detailed pre-enrollment and enrollment inclusion and exclusion criteria are included in the Supplementary Materials.

### Randomization, Intervention, and Blinding

Using a dynamic allocation method, patients were randomized at a ratio of 1:1 to receive placebo + PB (hereafter referred to as the placebo group) or tenapanor 5 mg twice daily (BID) + PB (hereafter referred to as the tenapanor group) for 8 weeks. Stratification factors were serum phosphorus level (≥6.1 mg/dl and ≤7.0 mg/dl, ≥7.1 mg/dl and ≤8.0 mg/dl, and ≥8.1 mg/dl and <10 mg/dl at enrollment) and study site. The placebo drug was indistinguishable from the study drug to ensure blinding. In addition, laboratory data on serum phosphorus levels, calcium-phosphate product, intact parathyroid hormone, intact fibroblast growth factor 23 (FGF23), and c-terminal FGF23 collected after the start of the study treatment were not reported to the investigator.

### Treatment and Dose Adjustments

Whereas a Japanese phase 2 study confirming the add-on effect of tenapanor to PBs used a tenapanor 30 mg down-titration regimen, the present study was designed to apply a new dose regimen, starting with tenapanor 5 mg BID, based on the efficacy and safety results of the Japanese phase 2 dose-response study.^18^

In this study, serum phosphorus levels (central laboratory values) were masked during the 8-week treatment period; however, but the laboratory company provided one of following alerts instead of reporting actual serum phosphorus levels: “10 mg/dl or greater: discontinue;” “6.1 mg/dl or greater: increase dose;” “4.5–6.0 mg/dl: dose can be increased;” “3.5–4.5 mg/dl: maintain dose;” and “less than 3.5 mg/dl: decrease dose.” The investigator adjusted the investigational product dose based on these alerts and the safety of the subjects. The details of the alerts and the background of the settings are provided in the Supplementary Materials. Each dose adjustment was performed immediately before meals following the completion of dialysis after the maximum dialysis interval. Drug interruption was allowed at any time, not only after the end of dialysis following the maximum dialysis interval.

After 2 weeks of tenapanor treatment, the dose was gradually increased in a stepwise manner using a 5 mg up-titration scheme (i.e., 5 mg–10 mg, 20 mg, or 30 mg BID) if all the dose adjustment criteria for dose increase were met and at least 2 weeks had elapsed since the latest dose adjustment. The criteria for dose increase were as follows: 1) the serum phosphorus level was ≥6.1 mg/dl after the latest maximum dialysis interval; or 2) the serum phosphorus level was ≥4.5 to ≤6.0 mg/dl, and the investigator considered that the dose could be increased for the patient. In addition, the investigator had to have considered that there were no safety concerns for the patient and that the dose could be increased.

For patients receiving the study drug at doses of ≥10 mg BID, the dose could be reduced in a stepwise manner if either of the following criteria were met: the serum phosphorus level was <3.5 mg/dl after the latest maximum dialysis interval or the investigator considered that the dose should be reduced because of the occurrence of gastrointestinal symptoms related to the study drug.

During the study, the dose, dosage form, type, or content of the existing PB could not be changed, except when a TEAE occurred for which a causal relationship with PB could not be ruled out, nor could a new dosage be taken. Several drugs and therapies were prohibited or restricted from concomitant use (Supplementary Materials).

### Study Outcomes

The primary end point was the change in serum phosphorus levels from baseline to 8 weeks after the start of tenapanor treatment. The secondary end points were the changes in serum phosphorus levels at each time point from baseline after administration of tenapanor and changes over time, as well as proportions of patients achieving target serum phosphorus level (3.5–6.0 mg/dl).

### Safety

The incidence of all TEAEs and adverse drug reactions that occurred or worsened after the start of the study treatment were summarized by treatment group using MedDRA (version 24.1). A TEAE was defined as any event that was not present before the start of the study treatment or any event already present that worsened in either intensity or frequency after the start of the study treatment. Laboratory parameters and vital signs were also evaluated.

### Data Collection

Data on the following test items were collected: clinical laboratory tests, serum phosphorus level, serum calcium level, intact parathyroid hormone (every 2 weeks), and FGF23 (week 0 and week 8) before the start of dialysis after the maximum dialysis interval. Patients collected data regarding changes in stool characteristics per the Bristol Stool Form Scale score and stool frequency per week in a patient diary.

### Sample Size Calculations

The sample size calculation for this study was based on data from previous studies.^16^, ^17^, ^18^, ^19^ A total of 104 subjects (52 subjects per group) was required under the assumption of a mean difference between the 2 groups of −1.0 mg/dl, a standard deviation of 1.8 mg/dl, with a 2-sided significance level of 5%, and 80% power. This study’s target subject number was 70 patients per group and 140 patients in total, considering a 30% withdrawal rate.

### Statistical Analysis

The analysis populations in this study were the modified intention-to-treat and the safety analysis sets. The modified intention-to-treat analysis set included all randomized patients who received at least 1 dose of the study drug and had serum phosphorus level measurements since the beginning of the study treatment. The safety analysis set included all randomized subjects except those who had not received the study drug.

Categorical data were summarized as frequencies and percentages, and continuous data were summarized using mean ± SD and median and range (minimum and maximum). The mixed model of repeated measures was used for the primary end point to compare the changes from baseline serum phosphorus levels in the tenapanor group and placebo group at week 8. The mixed model of repeated measures included changes from baseline in serum phosphorus level as a response variable, and treatment groups, timepoints of efficacy evaluation, the interaction of treatment groups by visit, and baseline serum phosphorus level as covariates. Imputation methods were not used to replace missing data for any end point. For secondary end points, means and SDs were calculated for serum phosphorus levels and changes from baseline at each visit in each group. In addition, data on the proportion of patients who achieved the target serum phosphorus level at each visit were collected for each group. The level of significance used was 5% and was 2-sided. All statistical analyses were performed using SAS Version 9.4 (SAS Institute, Cary, NC).

## Results

### Patient Disposition

**Figure 2:**
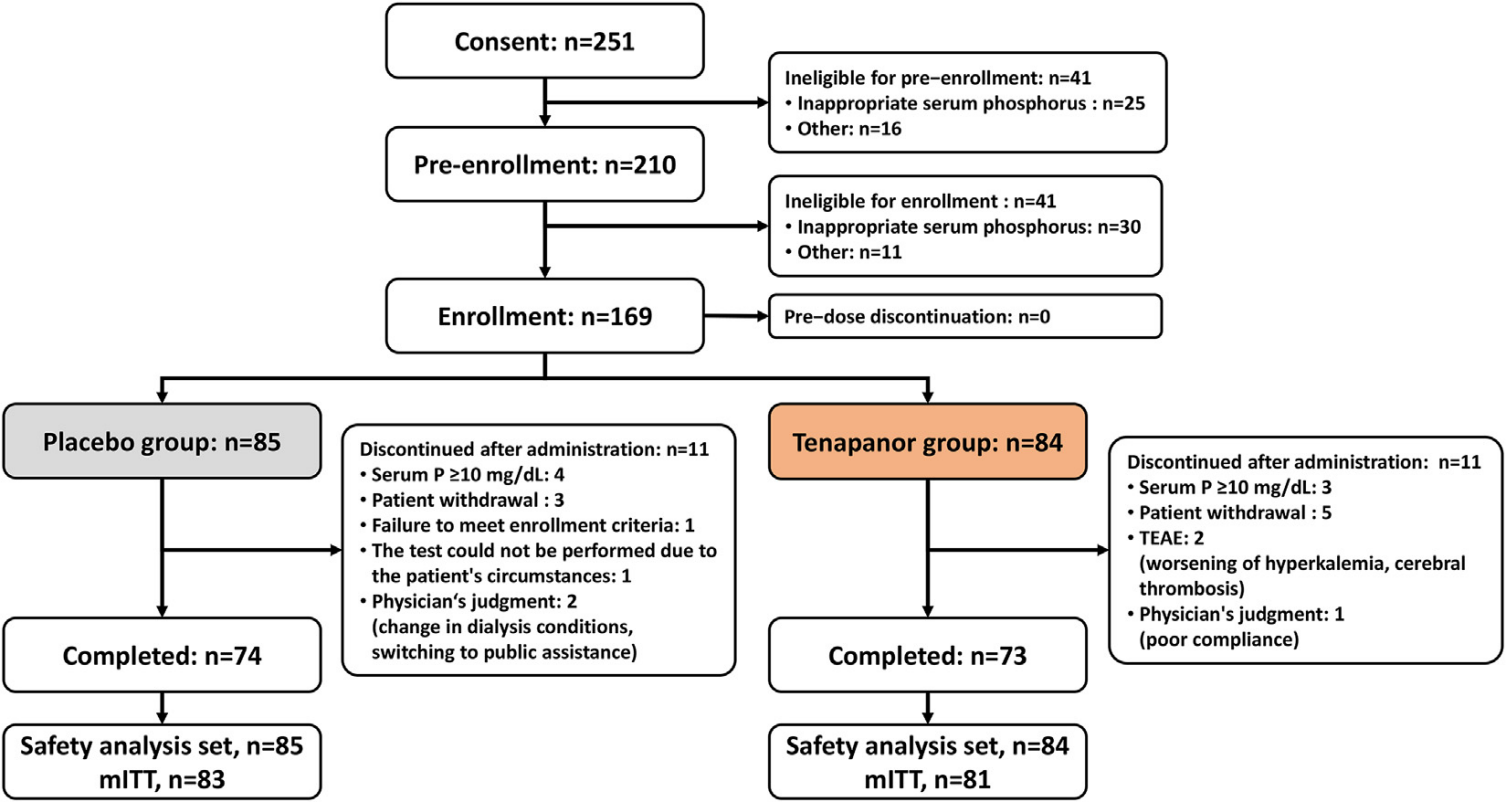
Patient disposition. ※ Tentative inclusion criteria 6) Patients with serum phosphorus levels of not less than 6.1 mg/dl and not more than 10.0 mg/dl at the preliminary examination Inclusion criteria 4) Patients with serum phosphorus levels of not less than 6.1 mg/dl and not more than 10.0 mg/dl at 1 or 2 weeks after the start of the run−in period. mITT, modified intention-to-treat; P, phosphorus; TEAE, treatment-emergent adverse event.

Of the 210 patients eligible for pre-enrollment, 41 were ineligible for enrollment. Of the 169 enrollment-eligible patients, all patients initiated the 8-week treatment period (placebo group, *n* = 85; tenapanor group, *n* = 84). After the treatment, 11 patients in each group discontinued the study, whereas 74 patients in the placebo group and 73 in the tenapanor group completed the treatment (Figure 2).

### Patient Characteristics

The baseline characteristics were generally similar between the groups (Table 1). In the placebo and tenapanor groups, 66.3% and 63.0% of patients were male and had a mean age of 60.6 years and 61.5 years, respectively. Diabetic nephropathy (30.1% and 38.3%) and chronic glomerulonephritis (32.5% and 38.3%) were the 2 most common underlying diseases in the placebo and tenapanor groups, respectively. Of the preexisting PBs at baseline, lanthanum carbonate (49.4% and 56.8%) and calcium carbonate (44.6% and 49.4%) were the most prescribed. The baseline serum phosphorus levels were 6.92 ± 1.068 mg/dl and 6.76 ± 1.075 mg/dl in the placebo and tenapanor groups, respectively.

**Table 1:** Baseline characteristics of patients in the modified intention-to-treat population

Background characteristic	Placebo + PB *n* = 83	Tenapanor + PB *n* = 81
Sex		
Female	28 (33.7%)	30 (37.0%)
Male	55 (66.3%)	51 (63.0%)
Age (mean ± SD, yrs)	60.6 ± 11.03	61.5 ± 11.19
<65 yrs	50 (60.2%)	46 (56.8%)
≥65 yrs	33 (39.8%)	35 (43.2%)
Body weight (mean ± SD, kg)	67.5 ± 14.35	67.2 ± 13.50
Type of dialysis		
Hemodialysis	28 (33.7%)	19 (23.5%)
Hemodiafiltration	55 (66.3%)	62 (76.5%)
Dialysate calcium concentration (mEq/l)		
2.5	27 (32.5%)	24 (29.6%)
2.75	33 (39.8%)	32 (39.5%)
3.0	23 (27.7%)	25 (30.9%)
Kt/V urea (mean ± SD)	1.55 ± 0.255	1.52 ± 0.225
nPCR (mean ± SD, g/kg/d)	0.96 ± 0.159	0.92 ± 0.212
Primary disease		
Diabetic nephropathy	25 (30.1%)	31 (38.3%)
Chronic glomerulonephritis	27 (32.5%)	31 (38.3%)
Nephrosclerosis	12 (14.5%)	4 (4.9%)
Polycystic kidney disease	8 (9.6%)	6 (7.4%)
Other	11 (13.3%)	9 (11.1%)
Phosphate binder at day 1		
Calcium carbonate	37 (44.6%)	40 (49.4%)
Sevelamer	11 (13.3%)	10 (12.3%)
Lanthanum carbonate	41 (49.4%)	46 (56.8%)
Bixalomer	14 (16.9%)	9 (11.1%)
Sucroferric oxyhydroxide	12 (14.5%)	13 (16.0%)
Ferric citrate	23 (27.7%)	22 (27.2%)
Use of PBs at baseline		
One type	38 (45.8%)	32 (39.5%)
Two or more types	45 (54.2%)	49 (60.5%)
Serum phosphorus level (mean ± SD, mg/dl)	6.92 ± 1.068	6.76 ± 1.075
Use of antidiarrheal drugs	6 (7.2%)	2 (2.5%)
Laxatives used	33 (39.8%)	32 (39.5%)
BSFS score (mean ± SD)	4.03 ± 0.952	3.95 ± 0.921
Stool frequency (mean ± SD, times/wk)	9.1 ± 3.77	8.4 ± 3.97

BSFS, Bristol Stool Form Scale; Kt/V urea, fractional urea clearance; nPCR, normalized protein catabolic rate; PB, phosphate binder; wk, week.

Data represent *n* (%) or mean ± SD.

### Study End Points

#### Primary End Point

**Figure 3:**
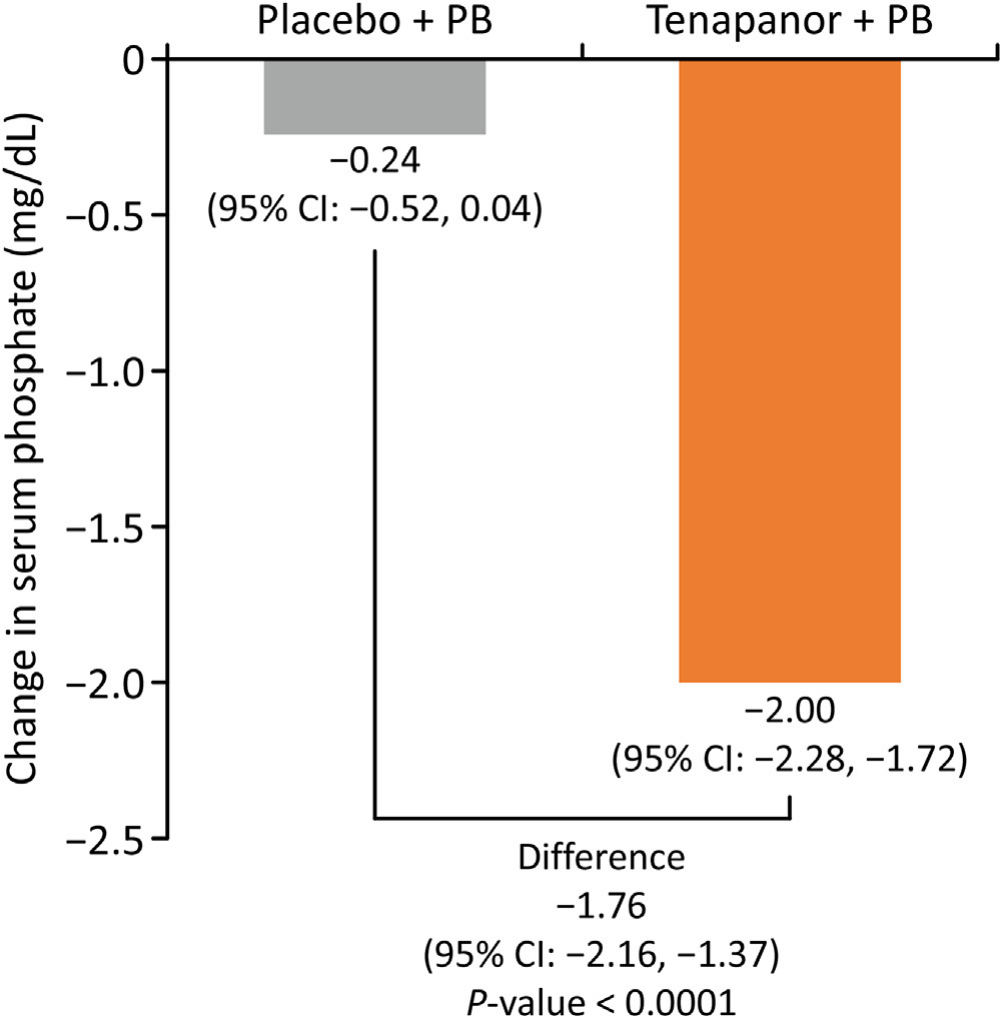
Change from baseline to week 8 in serum phosphorus level in each group. CI, confidence interval; PB, phosphate binder.

The least squares mean change from baseline to week 8 in serum phosphorus level was −0.24 mg/dl (95% confidence interval −0.52 to 0.04 mg/dl) in the placebo group and −2.00 mg/dl (95% confidence interval −2.28 to −1.72 mg/dl) in the tenapanor group, with a statistically significant difference between groups of −1.76 mg/dl (95% confidence interval −2.16 to −1.37 mg/dl) (*P* < 0.0001) (Figure 3).

**Figure 4:**
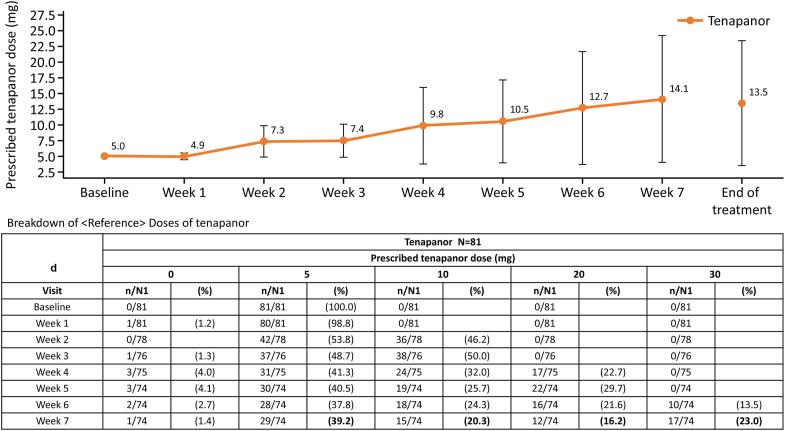
Change in the average dose of tenapanor (mg). *n*, number of subjects who were prescribed specified dose; N1, number of subjects who were prescribed a dose; week 0, baseline. Error bars represent standard deviation.

The tenapanor dose was up-titrated throughout the study, reaching a mean dose of 14.1 mg at week 7, the last prescription visit. At week 7, 39.2% of patients received tenapanor 5 mg BID; 20.3% received 10 mg BID; 16.2% received 20 mg BID; and 23.0% received 30 mg BID (Figure 4).

#### Secondary End Points

**Figure 5:**
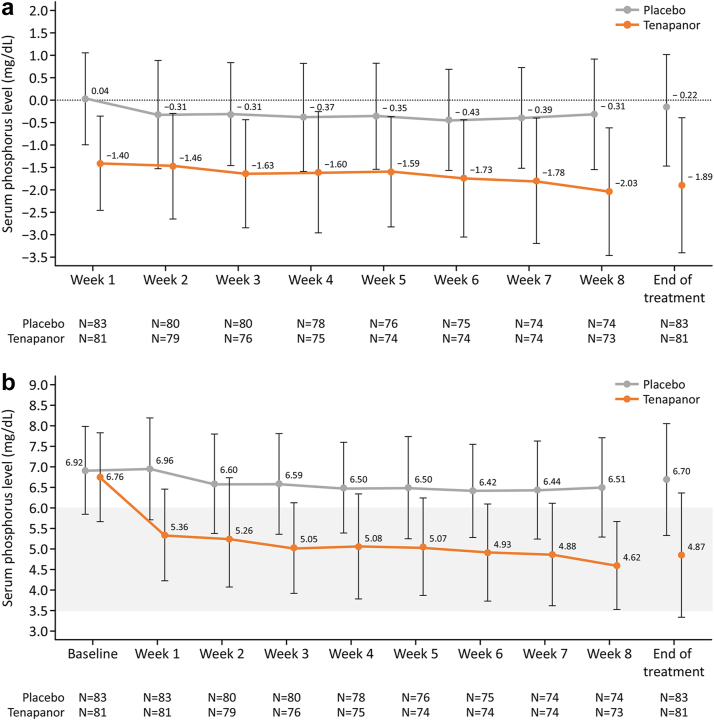
(a) Mean change from baseline in serum phosphorus level at each time point (mg/dl) and (b) time course of serum phosphorus level (mean) (mg/dl). Error bars represent standard deviation.

Although the changes in serum phosphorus levels over time remained constant in the placebo group from week 1 to week 8 (from 0.04 to −0.31 mg/dl), serum phosphorus levels in the tenapanor group decreased markedly from week 1 to week 8 (from −1.40 to −2.03 mg/dl). Serum phosphorus levels remained relatively constant in the placebo group (6.92 mg/dl at baseline to 6.51 mg/dl at week 8) but decreased markedly in the tenapanor group (6.76 mg/dl at baseline to 4.62 mg/dl at week 8; Figure 5a and b). In the tenapanor group, the higher the baseline serum, the greater the decrease in serum phosphorus levels (Supplementary Figure S1 and Supplementary Table S1).

**Figure 6:**
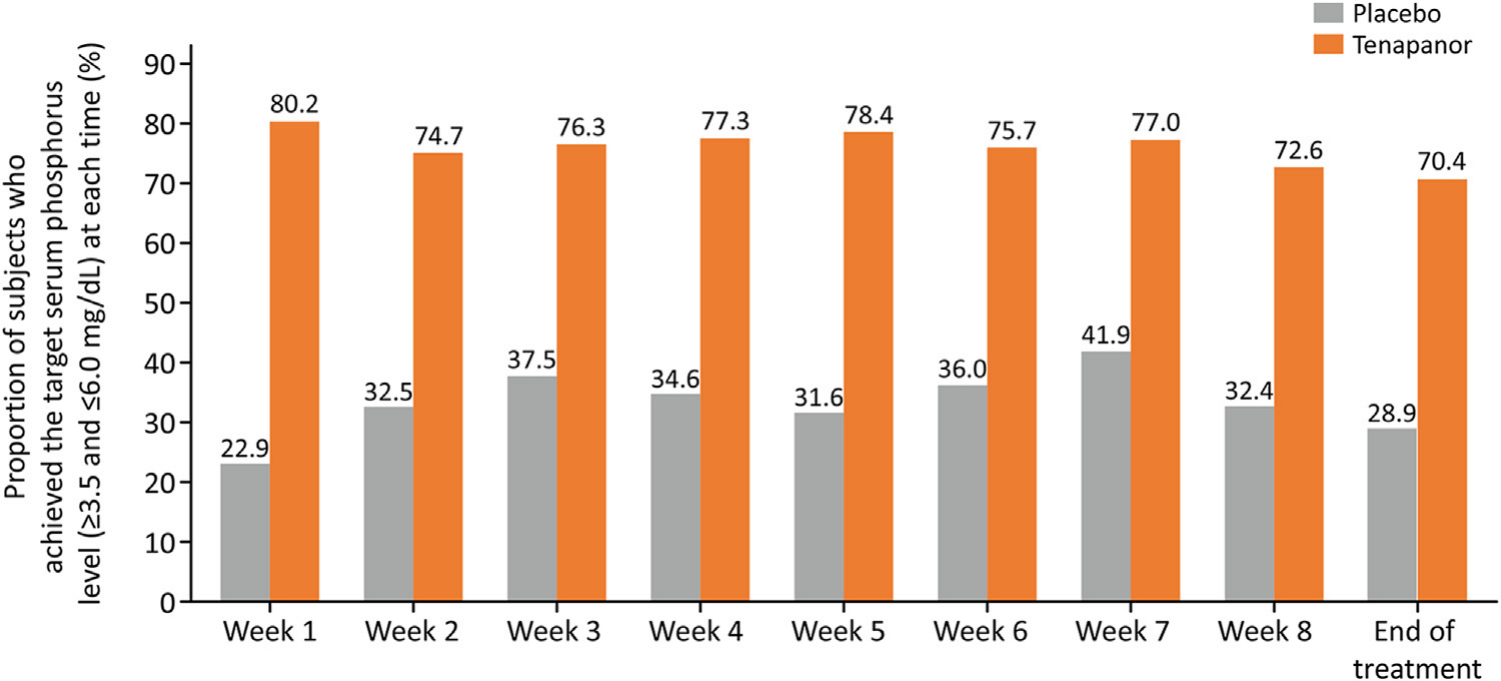
Serum phosphorus level of ≥3.5 mg/dl and ≤6.0 mg/dl at each time point.

In Figure 6, we show the proportions of patients achieving the target serum phosphorus of 3.5 to 6.0 mg/dl. The proportions of patients achieving the target serum phosphorus levels remained relatively consistent throughout the study in the placebo and tenapanor groups (22.9%–32.4% and 80.2%–72.6%, respectively). Because a certain number of patients in the tenapanor group had serum phosphorus levels below 3.5 mg/dl in each visit, the proportion of patients with serum phosphorus levels below 6.0 mg/dl remained around 80.0% and was 87.7% at week 8 (Supplementary Figure S2).

#### Stratified Analysis of Serum Phosphorus Level by PB Type

Adding tenapanor to the existing PBs had a similar serum phosphorus-lowering effect with each PB. Changes from baseline in serum phosphorus levels appeared to be more marked in the tenapanor group than in the placebo group at any time (Supplementary Figure S3).

#### Other Laboratory Parameters

The results of other parameters measured, such as laboratory data and bone metabolism markers, are described in Supplementary Figure S4 and Supplementary Table S2. The treatment did not have a significant effect on any bone metabolism marker. No significant changes were observed for other laboratory values, except intact FGF23 and intact parathyroid hormone. Intact FGF23 and intact parathyroid hormone did not change significantly from baseline in the placebo group but decreased from baseline in the tenapanor group.

#### Safety

During the study, TEAEs occurred in 62.4% (53/85) of patients in the placebo group and 85.7% (72/84) of patients in the tenapanor group. Diarrhea occurred in 14.1% (12/85) of patients in the placebo group and 63.1% (53/84) of patients in the tenapanor group. All events of diarrhea were mild or moderate in severity. In the tenapanor group, 75.5% (40/53) of diarrhea events were mild, and 24.5% (13/53) were moderate (Table 2).

**Table 2:** Treatment-emergent adverse events with an incidence >5% in any group

Event	Placebo + PB *n* = 85		Tenapanor + PB *n* = 84	
	*n*	(%)	*n*	(%)
TEAE with an incidence >5% in any group	53	(62.4)	72	(85.7)
Diarrhea	12	(14.1)	53	(63.1)
Severity of diarrhea^a^				
Mild	9	(75.0)	40	(75.5)
Moderate	3	(25.0)	13	(24.5)
Severe	0	(0.0)	0	(0.0)
Pyrexia	7	(8.2)	11	(13.1)
Pain in extremity	5	(5.9)	4	(4.8)
Serious adverse events	3	(3.5)	2	(2.4)
Death	0	(0.0)	0	(0.0)
Discontinuation				
Adverse events	0	(0.0)	2	(2.4)

PB, phosphate binder; TEAE, treatment-emergent adverse event.

a The severity was determined by the attending physician according to the following definitions: Mild: signs or symptoms present but not interfering with daily activities; Moderate: interferes with daily activities due to discomfort or affects the clinical status; Severe: inability to engage in daily activities or significant impact on clinical status.

The proportions of patients withdrawn from the study after initiating study treatment were 11 (12.9%) and 11 (13.1%) in the placebo and tenapanor groups, respectively. Two patients in the tenapanor group discontinued due to worsening hyperkalemia or cerebral thrombosis. No patients discontinued the study because of diarrhea as a TEAE.

**Figure 7:**
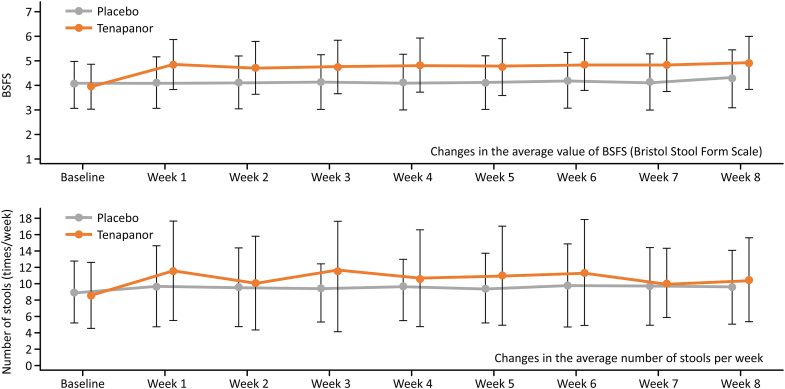
Mean change in BSFS from baseline. BSFS, Bristol Stool Form Scale. Error bars represent standard deviation.

The mean Bristol Stool Form Scale score remained consistent in the placebo group; however, in the tenapanor group, it increased (from 3.95 to 4.85) during week 1 by approximately 1 and remained relatively constant throughout the study. Similarly, the number of stools per week remained consistent in the placebo group, whereas, in the tenapanor group, it increased during week 1 and remained consistent thereafter (Figure 7).

Of the existing PBs associated with constipation as a TEAE, the incidence of diarrhea was lower with sevelamer and higher with bixalomer compared with the overall incidence of diarrhea in the tenapanor group. Among the iron-containing PBs that cause diarrhea, diarrhea frequency was higher with ferric citrate and lower with sucroferric oxyhydroxide compared with the overall frequency of diarrhea in the tenapanor group (Supplementary Table S3).

## Discussion

The present phase 3, placebo-controlled, randomized, parallel-group study demonstrates that tenapanor + PB significantly reduced serum phosphorous levels in patients with refractory hyperphosphatemia undergoing hemodialysis. The mean change from baseline in serum phosphorus levels in the tenapanor group was greater than in the placebo group, with a mean difference between groups of −1.76 mg/dl (95% confidence interval −2.16 to −1.37; *P* < 0.0001) at week 8. The present results showed that the administration of tenapanor, which has a novel mechanism of action, and was added to PBs, might exhibit an additional phosphorus-lowering effect in patients with poor serum phosphorus control with existing PBs.

The change from baseline to week 8 in serum phosphorus level in the tenapanor group in the present study (−2.00 mg/dl) was similar to that observed in a Japanese phase 3 study of tenapanor monotherapy for hemodialysis patients whose serum phosphorus levels were adequately controlled with existing PBs (−1.89 mg/dl).^20^ Of note, the characteristics of patients in the present study differ from those in the monotherapy study. The results suggest that tenapanor has a particular effect regardless of whether existing PBs are effective. This effect is probably due to its mechanism of action, which differs from that of existing PBs.

The mean change from baseline in serum phosphorus level was −1.40 mg/dl at week 1 in the tenapanor group. In addition, in the tenapanor group, the percentage of patients who achieved the target serum phosphorus level was as high as 80.2% as early as week 1 of tenapanor administration. All patients in the tenapanor group were taking tenapanor 5 mg BID at week 1, indicating the effectiveness of the 5 mg BID dose. Furthermore, the mean change from baseline in serum phosphorus level in the tenapanor group continued to increase throughout the study period from −1.40 mg/dl at week 1 to −2.03 mg/dl at week 8. These results may be related to the increase in the dose of tenapanor after week 2, suggesting that increasing the dose from 5 mg BID is also effective for treatment. Regarding the change in serum phosphorus level throughout this study and the dose distribution of tenapanor at week 7, the optimal dose of tenapanor varied from patient to patient, with some patients responding at the initial dose and others benefiting from an increased dose.

Although serum phosphorus levels in the tenapanor group decreased with increasing tenapanor dose, the percentage of subjects who achieved the target serum phosphorus level remained relatively constant from week 1 (80.2%) to week 8 (72.6%) in the tenapanor group. This result was attributable to the fact that in a certain number of patients, serum phosphorus levels fell below the lower limit of the target serum phosphorus level (3.5 mg/dl) at each visit. We consider this to be the result of tenapanor being too effective for some patients even at the lowest dose of 5 mg, and serum phosphorus levels becoming too low in some patients despite the investigator increasing the dose of tenapanor based on dose adjustment criteria. In addition, a number of patients in the tenapanor group could control serum phosphorus levels within the range of 3.5 to 4.5 mg/dl in this study. A recent study reported that a stricter serum phosphorus control range (3.5–4.5 mg/dl) was associated with delayed coronary artery calcification, which could translate into a lower risk of cardiovascular mortality.^9^ Therefore, even if serum phosphorus levels are poorly controlled with existing PBs, adding tenapanor to PBs may contribute to reducing the risk of vascular calcification. The proportion of patients achieving the target range in the placebo group was slightly larger. However, the serum phosphorus levels in the placebo group did not decrease throughout the study. In addition, a breakdown of the serum phosphorus levels of these patients shows that most of them had serum phosphorus levels between 4.5 and 6.0 mg/dl (Supplementary Figure S2). From these results, the larger percentage of patients who achieved the target range in the placebo group may be due to physiological variations in serum phosphorus levels in each patient.

It was also found that the higher the baseline serum phosphorus level, the more tenapanor reduced serum phosphorus levels, suggesting that the more poorly controlled the serum phosphorus levels are with existing PBs alone, the greater the add-on effect of tenapanor that can be expected.

Approximately 63% of patients in the tenapanor group experienced diarrhea, which is consistent with the mechanism of action of tenapanor. Over 75% of these events were considered mild, and over 24% were considered moderate in severity. The Bristol Stool Form Scale score and the number of bowel movements per week increased immediately after the start of tenapanor administration and remained at similar levels after week 2. This is consistent with the finding that, although the incidence of diarrhea was high, its severity was relatively mild. Further, the proportions of patients who withdrew from the study and reasons for withdrawal were similar in both groups, and none of the patients discontinued or withdrew from the study because of diarrhea as a TEAE.

Many dialysis patients have constipation as a complication.^21^^,^^22^ Some of these patients may receive laxatives, further increasing their pill burden. In the present study, 33 (39.8%) patients in the placebo group and 32 (39.5%) patients in the tenapanor group were using laxatives before study initiation. Although diarrhea as a TEAE was reported by several patients in this study, this effect of tenapanor may help relieve constipation. In that respect, the administration of tenapanor may further decrease the pill burden by decreasing the need for laxative medications.

The 2 serious TEAEs reported in the present study (multiple colorectal polyps and cerebral thrombosis) were considered unrelated to tenapanor treatment. Therefore, as reported in the Japanese study evaluating the effects of add-on tenapanor for hemodialysis patients,^17^ the primary TEAE was diarrhea. The new dosage regimen and PB combinations raised no other safety concerns, suggesting that tenapanor was safe and well tolerated.

Regarding the pattern of combinations with PBs, the efficacy of tenapanor was similar in combination with each PB regardless of the PB type. This result may be due to the additive effect of the novel mechanism of action of tenapanor with that of PBs. As for safety, no trend was observed in the frequency of diarrhea when tenapanor was combined with PBs that cause constipation or diarrhea as a TEAE.

The decrease in intact FGF23 levels in the tenapanor group observed in this study was similarly observed in the US phase 3 and Japanese phase 2 studies. Although the mechanism of action is not clear, intact FGF23 is located upstream of the regulation of serum phosphorus level in humans.^23^ Therefore, it is possible that the decrease in serum phosphorus levels caused by tenapanor treatment also contributed to the decrease in intact FGF23. Thus, tenapanor could lead to a reduction in cardiovascular disease risk.

Data on dietary intake were not collected in this study. Considering that the administration of tenapanor improved phosphorus control, future studies may reveal whether it would be possible to relax dietary restrictions during tenapanor administration.

### Limitations

This study’s main limitation was the short treatment duration (8 weeks). The efficacy and tolerability of long-term treatment with tenapanor combined with PBs for patients with hemodialysis should therefore be verified in other studies. In the present study, more than half of the patients used more than 1 type of PB; therefore, further research focusing on the efficacy and safety of individual PBs may bring new insights. Other limitations of this study include non-generalizability to other regions, where most PBs are calcium-based or sevelamer rather than lanthanum carbonate or ferric citrate, and the high proportion of patients receiving hemodiafiltration dialysis.

### Conclusions

The administration of tenapanor + PB to Japanese patients with hyperphosphatemia undergoing hemodialysis who had poorly controlled serum phosphorus levels resulted in a significant reduction in serum phosphorus levels and an increased proportion of patients achieving the target serum phosphorus levels compared with the placebo + PB group. No new safety concerns were raised, and all diarrhea events were either mild or moderate. The discontinuations for TEAEs were similar in the tenapanor and placebo groups. Therefore, tenapanor, added to other PBs, was safe and tolerable for hemodialysis patients with hyperphosphatemia in Japan. The present findings suggest that tenapanor combined with other PBs could be a treatment option for patients undergoing hemodialysis who have poor control of serum phosphorus levels with existing PBs.

## Disclosure

KN reports personal fees from Kyowa Kirin Co. Ltd. during the conduct of the study and grants and personal fees from Kyowa Kirin Co. Ltd. outside the submitted work. TA reports personal fees from Kyowa Kirin Co. Ltd. during the conduct of the study; personal fees from Ono pharmaceutical Co. Ltd., personal fees from Sanwa Kagaku Kenkyusyo Co. Ltd., grants and personal fees from Kyowa Kirin Co. Ltd., personal fees from Bayer Yakuhin Ltd., personal fees from Kissei Pharmaceutical Co. Ltd., personal fees from Torii Pharmaceutical Co. Ltd., and personal fees from Astellas Pharma Inc., outside the submitted work. MF reports personal fees from Kyowa Kirin Co. Ltd. during the conduct of the study; personal fees from Ono pharmaceutical Co. Ltd., personal fees from Sanwa Kagaku Kenkyusyo Co. Ltd., personal fees from Kyowa Kirin Co. Ltd., personal fees from Bayer Yakuhin Ltd., and personal fees from Kissei Pharmaceutical Co. Ltd. outside the submitted work. SI, KI, JK, and KN are employees of Kyowa Kirin.

## Supplementary Material

**Supplementary Methods.**

**Supplementary Results.**

**Figure S1.** Change in serum phosphorus levels by baseline serum phosphorus level

**Figure S2.** Breakdown of the proportion of patients by serum phosphorus levels over time (a) in the placebo group and (b) the tenapanor group.

**Figure S3.** Change in serum phosphorus level by phosphate binder type.

**Figure S4.** Change in bone metabolism markers.

**Table S1.** Change from Baseline in Serum Phosphorus at Week 8 by baseline serum phosphorus level.

**Table S2.** Change in laboratory parameters and bone metabolism markers.

**Table S3.** Frequency of drug-related TEAEs by type of PB added to tenapanor (adverse event with an incidence >5% and more than 1 patient in any group.

## References

[1] Young E.W., Albert J.M., Satayathum S. (2005). Predictors and consequences of altered mineral metabolism: the Dialysis Outcomes and Practice Patterns Study. Kidney Int.

[2] N. Kimata, J.M. Albert, T. Akiba, et al., Association of mineral metabolism factors with all-cause and cardiovascular mortality in hemodialysis patients: the Japan dialysis outcomes and practice patterns study, *Hemodial Int*, **11**, 2007, 340–348, doi: 10.1111/j.1542-4758.2007.00190.x. 10.1111/j.1542-4758.2007.00190.x 17576300

[3] Shaman A.M., Kowalski S.R. (2016). Hyperphosphatemia management in patients with chronic kidney disease. Saudi Pharm J.

[4] Cozzolino M., Mangano M., Stucchi A., Ciceri P., Conte F., Galassi A. (2018). Cardiovascular disease in dialysis patients. Nephrol Dial Transplant.

[5] Tentori F., Blayney M.J., Albert J.M. (2008). Mortality risk for dialysis patients with different levels of serum calcium, phosphorus, and PTH: the Dialysis Outcomes and Practice Patterns Study (DOPPS). Am J Kidney Dis.

[6] Floege J., Kim J., Ireland E. (2011). Serum iPTH, calcium and phosphate, and the risk of mortality in a European haemodialysis population. Nephrol Dial Transplant.

[7] (2012). The Japanese Society for Dialysis Therapy. Clinical practice guideline for CKD-MBD. Nihon Toseki Igakkai Zasshi.

[8] National Kidney Foundation (2003). K/DOQI clinical practice guidelines for bone metabolism and disease in chronic kidney disease. Am J Kidney Dis.

[9] Isaka Y., Hamano T., Fujii H. (2021). Optimal phosphate control related to coronary artery calcification in dialysis patients. J Am Soc Nephrol.

[10] Barreto F.C., Barreto D.V., Massy Z.A., Drüeke T.B. (2019). Strategies for phosphate control in patients with CKD. Kidney Int Rep.

[11] Fissell R.B., Karaboyas A., Bieber B.A. (2016). Phosphate binder pill burden, patient-reported non-adherence, and mineral bone disorder markers: findings from the DOPPS. Hemodial Int.

[12] Wang S., Alfieri T., Ramakrishnan K., Braunhofer P., Newsome B.A. (2014). Serum phosphorus levels and pill burden are inversely associated with adherence in patients on hemodialysis. Nephrol Dial Transplant.

[13] Van Camp Y.P., Vrijens B., Abraham I., Van Rompaey B., Elseviers M.M. (2014). Adherence to phosphate binders in hemodialysis patients: prevalence and determinants. J Nephrol.

[14] Spencer A.G., Labonte E.D., Rosenbaum D.P. (2014). Intestinal inhibition of the Na+/H+ exchanger 3 prevents cardiorenal damage in rats and inhibits Na+ uptake in humans. Sci Transl Med.

[15] Labonté E.D., Carreras C.W., Leadbetter M.R. (2015). Gastrointestinal inhibition of sodium-hydrogen exchanger 3 reduces phosphorus absorption and protects against vascular calcification in CKD. J Am Soc Nephrol.

[16] Pergola P.E., Rosenbaum D.P., Yang Y., Chertow G.M. (2021). A randomized trial of tenapanor and phosphate binders as a dual-mechanism treatment for hyperphosphatemia in patients on maintenance dialysis (AMPLIFY). J Am Soc Nephrol.

[17] Shigematsu T., Une Y., Ikejiri K., Kanda H., Fukagawa M., Akizawa T. (2021). Therapeutic effects of add-on tenapanor for hemodialysis patients with refractory hyperphosphatemia. Am J Nephrol.

[18] Inaba M., Une Y., Ikejiri K., Kanda H., Fukagawa M., Akizawa T. (2021). Dose-response of tenapanor in patients with hyperphosphatemia undergoing hemodialysis in Japan-a phase 2 randomized trial. Kidney Int Rep.

[19] Block G.A., Rosenbaum D.P., Yan A., Chertow G.M. (2019). Efficacy and safety of tenapanor in patients with hyperphosphatemia receiving maintenance hemodialysis: a randomized phase 3 trial. J Am Soc Nephrol.

[20] Fukagawa M., Urano N., Ikejiri K., Kinoshita J., Nakanishi K., Akizawa T. (2023). Tenapanor for the treatment of hyperphosphatemia in Japanese hemodialysis patients: a randomized phase 3 monotherapy study with an “UP-titration” regimen. Am J Kidney Dis.

[21] Cano A.E., Neil A.K., Kang J.Y. (2007). Gastrointestinal symptoms in patients with end-stage renal disease undergoing treatment by hemodialysis or peritoneal dialysis. Am J Gastroenterol.

[22] Ikee R., Yano K., Tsuru T. (2019). Constipation in chronic kidney disease: it is time to reconsider. Ren Replace Ther.

[23] Quarles L.D. (2008). Endocrine functions of bone in mineral metabolism regulation. J Clin Invest.

