# Supplementary material for: Randomized Study of Tenapanor Added to Phosphate Binders for Patients With Refractory Hyperphosphatemia
Source: Kidney Int Rep. 2023 Aug 13;8(11):2243–53. doi: 10.1016/j.ekir.2023.08.003 (PMC10658421; doi:10.1016/j.ekir.2023.08.003)
Supplement: Supplementary File (PDF) [file mmc1.pdf]

SUPPLEMENTARY MATERIALS

Supplementary Methods

Inclusion criteria for pre-enrollment

Patients were eligible for pre-enrollment if they met the following criteria:

1. Voluntarily provided informed consent to participate in the study
2. ≥20 years of age
3. Patients with stable chronic renal failure who have undergone hemodialysis three times per week for at least 12 weeks until screening examination
4. Dialysis conditions (dialysate, dialyzer, frequency of dialysis per week, dialysis duration, blood flow rate, and dialysate and substitution fluid flow rates), excluding dry weight, should have been unchanged during the last 2 weeks before screening
5. Taking phosphate binders with the dosing regimen unchanged during the last 2 weeks
6. Serum phosphorus levels should be in the range of ≥6.1 mg/dL to <10.0 mg/dL at screening examination
7. If on treatment with vitamin D supplementation, calcimimetics, bisphosphonates, calcitonin, selective estrogen receptor modulators, or teriparatide, the prescribed dosing regimen should have been unchanged for the last 2 weeks before screening examination
8. Single pool Kt/V urea ≥1.2 at the most recent test in routine medical practice before screening examination.

Exclusion criteria for pre-enrollment

Patients were ineligible for pre-enrollment if they met any of the following criteria:

1. Had undergone concomitant peritoneal dialysis within 12 weeks before screening examination

2. Intact parathyroid hormone >600 pg/mL at screening examination
3. Concurrent or history of inflammatory bowel disease or diarrhea-predominant irritable bowel syndrome
4. History of gastrectomy or enterectomy (excluding endoscopic resection and appendectomy) or having undergone gastrointestinal tract surgery (excluding endoscopic resection and appendectomy) within 3 months before screening examination
5. Received anti-RANKL antibody preparations within 6 weeks before screening examination
6. Received anti-sclerostin antibody preparations within 12 weeks before screening examination
7. Concurrent severe heart disease (including congestive heart failure, defined as New York Heart Association cardiac functional class III or IV, and vascular lesions requiring hospitalization, such as myocardial infarction) or hepatic impairment (including aspartate aminotransferase/alanine aminotransferase ≥100 U/L at screening examination, or cirrhosis)
8. Developed cerebrovascular disease (such as cerebral infarction and hemorrhage) or cardiovascular disease (such as myocardial infarction and unstable angina) requiring hospitalization within 6 months before screening examination
9. Uncontrollable hypertension or diabetes
10. Scheduled for living donor kidney transplant, change in the mode of dialysis, home hemodialysis, or change in the dialysis center (relocate to another hospital/clinic) during the study period
11. Any diagnosis of and treatment of malignancy within 5 years before screening examination (excluding basal cell carcinoma or surgically resected intraepithelial carcinoma of uterine cervix)
12. Tested positive for human immunodeficiency virus or human T-cell leukemia virus type 1

13. Expected to develop serious drug allergies, such as anaphylactic shock, or any history of alcohol dependence, illicit drug use, severe mental illness, or drug abuse or addiction within 12 months before screening examination
14. Not expected to live for ≥12 months
15. Received other study drugs within 4 weeks before screening examination (if a study drug used outside the above period may have affected the efficacy and safety assessments of tenapanor, for example, in the case that the 5-fold elimination half-life of the study drug exceeds 4 weeks, the eligibility of the subject should be discussed with the sponsor)
16. Previous exposure to tenapanor
17. Pregnant women or nursing mothers, or female patients with a desire to bear children
18. Female patients of childbearing potential who did not agree to use an adequate birth control method from the date of providing informed consent to 3 weeks after completion of study treatment or fertile male patients who did not agree to practice an adequate birth control method from the date of the start of study treatment to 3 weeks after completion of study treatment during the study period
19. Any medical or other condition that, in the opinion of the investigator, precluded participation in the study.

Inclusion criteria for enrollment

Patients were eligible to participate in the study if they met all of the following criteria:

1. Patients with stable chronic renal failure who continued to undergo hemodialysis three times per week since pre-enrollment
2. Dialysis conditions (dialysate, dialyzer, frequency of dialysis per week, dialysis duration, blood flow rate, and dialysate and substitution fluid flow rates), excluding dry weight, should have remained unchanged since pre-enrollment

3. Continued to receive phosphate binders with the same dosing regimen for the period from pre-enrollment through enrollment
4. Had serum phosphorus levels should be in the range of ≥6.1 mg/dL to <10.0 mg/dL 1 or 2 weeks after the start of the run-in period
5. If on treatment with vitamin D supplementation, calcimimetics, bisphosphonates, calcitonin preparations, selective estrogen receptor modulators, or teriparatide preparations, the prescribed dosing regimen should have remained unchanged from pre-enrollment through enrollment.

Exclusion criteria for enrollment

Patients were ineligible for enrollment if they met any of the following criteria:

1. Started concomitant peritoneal dialysis after pre-enrollment
2. Diagnosed with inflammatory bowel disease or diarrhea-predominant irritable bowel syndrome after pre-enrollment
3. Underwent gastrointestinal tract surgery, such as gastrectomy or enterectomy (excluding endoscopic resection and appendectomy) after pre-enrollment or was scheduled to undergo such surgery during the study period
4. Received anti-RANKL antibody preparations or anti-sclerostin antibody preparations after pre-enrollment
5. Found to have severe heart disease (including congestive heart failure, defined as New York Heart Association cardiac functional class III or IV, and vascular lesions requiring hospitalization, such as myocardial infarction) or hepatic impairment (including aspartate aminotransferase/alanine aminotransferase ≥100 U/L during the run-in period, or cirrhosis) after pre-enrollment

6. Developed cerebrovascular disease (such as cerebral infarction and hemorrhage) or cardiovascular disease (such as myocardial infarction and unstable angina) requiring hospitalization after pre-enrollment
7. Found to have uncontrollable hypertension or diabetes after pre-enrollment
8. Had diarrhea or loose stools, defined as Bristol Stool Form Scale score ≥6 and frequency ≥3 for ≥2 days within 1 week before enrollment
9. Newly scheduled for living donor kidney transplant, change in the mode of dialysis, home hemodialysis, or change in the dialysis center (relocate to another hospital/clinic) during the study period after pre-enrollment
10. Any diagnosis of malignancy or newly discovered concurrent malignancy after pre-enrollment (excluding basal cell carcinoma or surgically resected intraepithelial carcinoma of uterine cervix)
11. Tested positive for human immunodeficiency virus or human T-cell leukemia virus type 1 after pre-enrollment
12. Expected to develop serious drug allergies, such as anaphylactic shock after pre-enrollment, or a history of alcohol dependence, illicit drug use, severe mental illness, or drug abuse or addiction identified after pre-enrollment
13. Received other study drugs after pre-enrollment
14. Received tenapanor during the period after pre-enrollment and before enrollment
15. Female patients found to be pregnant or who desired to bear children during the study period after pre-enrollment
16. Female patients of childbearing potential who withdrew their consent to use an adequate birth control method from the date of providing informed consent to 3 weeks after completion of study treatment or fertile male patients who did not agree to practice an adequate birth control method from the date of the start of study treatment to 3 weeks after completion of study treatment

17. Any medical or other condition that, in the opinion of the investigator precluded participation in the study after pre-enrollment.

The background of the settings of the alerts of serum phosphorous levels

The investigator adjusted the investigational product dose based on alerts for serum phosphorous levels and the safety of the patients. Blinding was maintained because actual serum phosphorus levels were not reported to the investigators and serum phosphorus levels are variable. It was assumed that serum phosphorus levels are easily influenced by diet and other factors and that alerting the investigators to serum phosphorus levels in this study would not affect blinding. In fact, in the phase 2 study conducted in Japan in hemodialysis patients with refractory hyperphosphatemia, approximately 20%–50% of the placebo group had serum phosphorus levels within the target range (≤6.0 mg/dL) during the study period. In the current study, 20%–40% of patients on placebo were within the target range (3.5–6.0 mg/dL) during the study period (Figure S2).

Restricted drugs and therapies

The use of calcium preparations was allowed at bedtime only for calcium supplementation from the date of the screening examination to the end of the test at Week 8. The dosing regimen of active vitamin D preparations and their derivatives could not be changed for 2 weeks before the screening examination and during the study with the following exceptions: for corrected serum calcium levels >11.0 mg/dL after the start of study treatment, active vitamin D preparations or their derivatives could be reduced or discontinued. If active vitamin D preparations or their derivatives were required after dose reduction or discontinuation, the dose could be increased to the level at the start of the study treatment. The use of new vitamin D preparations or their derivatives or increasing their dose was allowed in either of the

following cases: corrected serum calcium levels ≤7.5 mg/dL or onset of clinical symptoms presumed to be due to hypocalcemia.

The dosing regimen of calcimimetics, bisphosphonates, calcitonin preparations, selective estrogen receptor modulators, and teriparatide preparations could not be changed for 2 weeks before the screening examination and during the study.

Any change in dialysis conditions (dialysate, dialyzer, frequency of dialysis per week, dialysis duration, blood flow rate, and dialysate and substitution fluid flow rates), excluding dry weight, and diet was prohibited for 2 weeks before screening examination and during the study.

Figure S1. Change in serum phosphorus levels by baseline serum phosphorus level

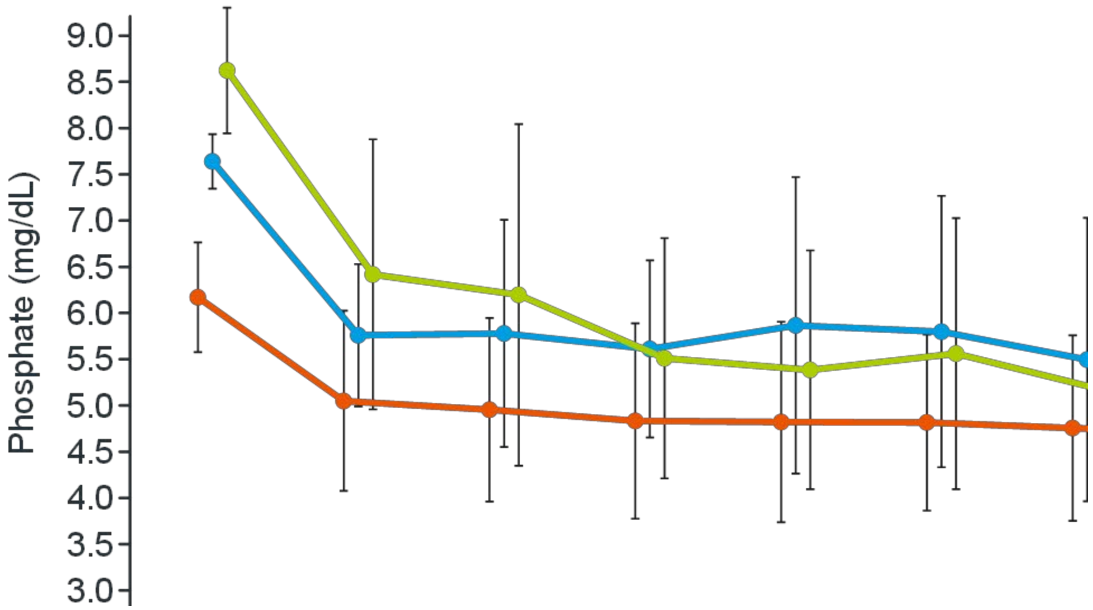

Figure S2. Breakdown of the proportion of patients by serum phosphorus levels over time in **(a)** the placebo group and **(b)** the tenapanor group

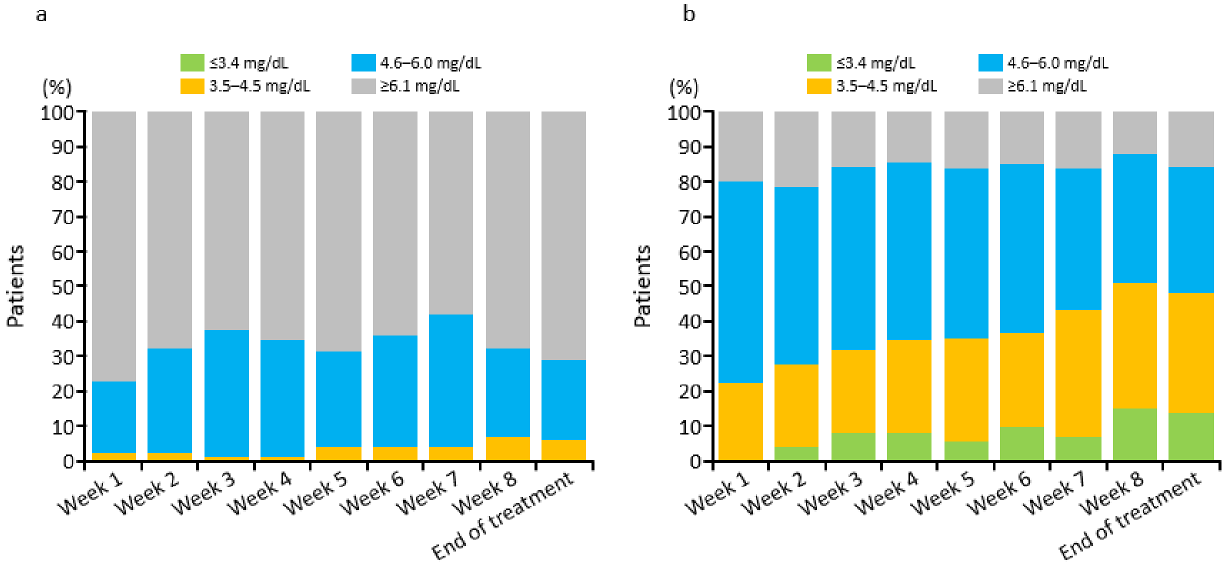

Figure S3. Change in serum phosphorus level by phosphate binder type

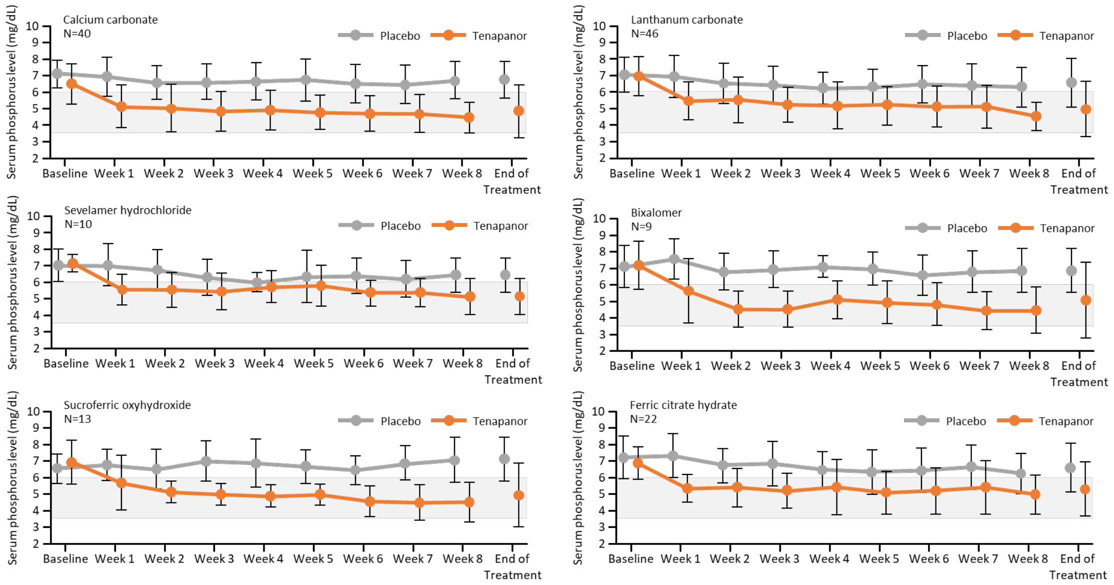

Figure S4. Change in bone metabolism markers

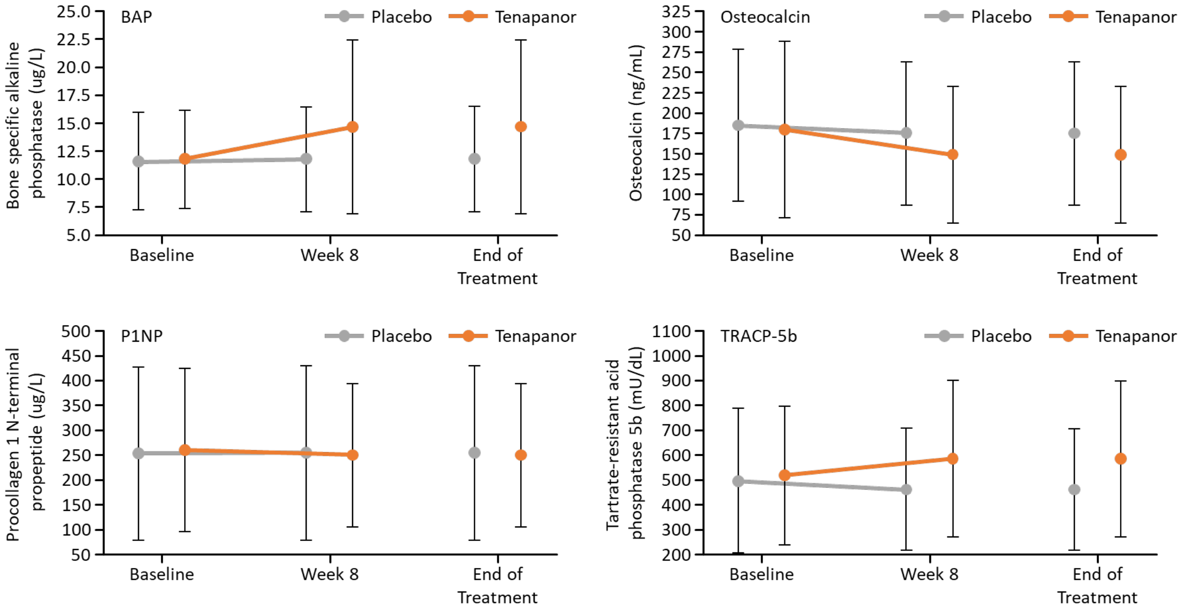

BAP, bone-specific alkaline phosphatase; P1NP, N-terminal propeptide of type I procollagen; TRACP-5b, tartrate resistant acid phosphatase 5b

Table S1. Change from baseline in serum phosphorus at week 8 by baseline serum phosphorus level

			Placebo N = 83	Tenapanor N = 81
**Serum phosphorus level at baseline (mg/dL)**	**<= 7.0**	N1	50	55
		n	46	52
		LS Mean	0.12	−1.58
		[95%CI]	[−0.18, 0.41]	[−1.86, −1.31]
		Difference		−1.70
		[95%CI]		[−2.10, −1.29]
		p-value		<.0001
	**>= 7.1 to <= 8.0**	N1	20	15
		n	19	13
		LS Mean	−0.89	−2.39
		[95%CI]	[−1.63, −0.14]	[−3.25, −1.52]
		Difference		−1.50
		[95%CI]		[−2.64, −0.36]
		p-value		0.0119
	**>= 8.1**	N1	13	11
		n	9	8
		LS Mean	−0.90	−3.77
		[95%CI]	[−1.70, −0.10]	[−4.63, −2.91]
		Difference		−2.87
		[95%CI]		[−4.05, −1.69]
		p-value		0.0002

N1: Number of subjects in the subpopulation at baseline

Table S2. Change in laboratory parameters and bone metabolism markers

	Placebo + PB	Tenapanor + PB
**Corrected Ca (mg/dL)**		
Baseline	9.22 ± 0.703	9.03 ± 0.665
Week 8	9.25 ± 0.654	9.19 ± 0.683
Variable amount	0.03 ± 0.398	0.16 ± 0.431
**Na (mEq/L)**		
Baseline	138.7 ± 2.51	138.6 ± 2.61
Week 8	138.8 ± 2.71	137.9 ± 2.70
Variable amount	0.0 ± 2.23	−0.8 ± 2.46
**K (mEq/L)**		
Baseline	5.10 ± 0.664	4.95 ± 0.676
Week 8	4.85 ± 0.594	4.99 ± 0.825
Variable amount	−0.24 ± 0.567	0.08 ± 0.700
**Cl (mEq/L)**		
Baseline	101.8 ± 3.02	101.4 ± 3.15
Week 8	101.9 ± 3.04	101.9 ± 2.89
Variable amount	0.0 ± 2.88	0.5 ± 2.55
**Mg (mg/dL)**		
Baseline	2.56 ± 0.375	2.47 ± 0.393
Week 8	2.51 ± 0.376	2.45 ± 0.386

Variable amount	−0.08 ± 0.172	−0.05 ± 0.257
**HCO_3^- (mmol/L)**		
Baseline	18.38 ± 2.169	18.52 ± 1.926
Week 8	18.46 ± 1.920	18.63 ± 2.322
Variable amount	0.02 ± 1.571	0.05 ± 1.977
**Intact FGF23 (pg/mL)**		
Baseline	14100.00 (427.0, 80000.0)	7080.00 (185.0, 80000.0)
Week 8	10700.00 (251.0, 62300.0)	3440 (165.0, 8000.0)
Variable amount	−88.50 (-40100.0, 21300.0)	−2690.00 (-44600.0, 10500.0)
**Intact PTH (pg/mL)**		
Baseline	172.0 (34, 813)	168.0 (17, 659)
Week 8	178.5 (35, 876)	136.0 (13, 622)
Variable amount	-1.0 (-132, 360)	−29.0 (-185, 67)

Data are presented as mean ± SD or median (min, max).

Ca, calcium; Cl, chloride; HCO_3^-, bicarbonate; FGF23, fibroblast growth factor 23; K, potassium; Mg, magnesium; Na, sodium; PTH, parathyroid hormone; SD, standard deviation.

Corrected Ca, intact PTH and intact FGF23 were analyzed with the modified intention-to-treat (Placebo + PB: n=83, Tenapanor + PB: n=81), and other parameters were analyzed with safety analysis set (Placebo + PB: n=85, Tenapanor + PB: n=84).

Table S3. Frequency of drug-related TEAEs by type of PB added to Tenapanor (adverse event with an incidence >5% and more than one patient in any group)

	Placebo N = 85						
	Total N = 85	Calcium carbonate n = 38	Sevelamer n = 11	Lanthanum carbonate n = 42	Bixalomer n = 14	Sucroferric oxyhydroxide n = 12	Ferric citrate n = 24
	n (%)	n (%)	n (%)	n (%)	n (%)	n (%)	n (%)
**Drug-related TEAEs**	12 (14.1)	4 (10.5)	2 (18.2)	9 (21.4)	0	1 (8.3)	4 (16.7)
Diarrhea	8 (9.4)	2 (5.3)	0	7 (16.7)	0	0	3 (12.5)
Abdominal discomfort	2 (2.4)	1 (2.6)	1 (9.1)	1 (2.4)	0	1 (8.3)	0
	**Tenapanor** N = 84						
	Total N = 84	Calcium carbonate	Sevelamer N = 11	Lanthanum carbonate	Bixalomer n = 10	Sucroferric oxyhydroxide	Ferric citrate n = 22

		n = 40		n = 49		n = 13	
**Drug-related TEAEs**	54 (64.3)	27 (67.5)	6 (54.5)	33 (67.3)	7 (70.0)	6 (46.2)	16 (72.7)
Diarrhea	49 (58.3)	25 (62.5)	4 (36.4)	29 (59.2)	7 (70.0)	5 (38.5)	16 (72.7)
Soft stool	4 (4.8)	2 (5.0)	2 (18.2)	2 (4.1)	0	0	0
Nausea	2 (2.4)	2 (5.0)	0	1 (2.0)	1 (10.0)	0	1 (4.5)

TEAE, treatment-emergent adverse event; PB, phosphate binder
